# Frutalin as an Affinity Tool for sIgA1: Biophysical and Structural Characterization of the Lectin–Antibody Complex

**DOI:** 10.1021/acsomega.5c06812

**Published:** 2025-12-05

**Authors:** Roberta C. C. Costa, Talita A. Leite, José G. S. Gomes, Francisco P. F. Silva, Marcus R. L. Bezerra, Wallady S Barroso, Gilvan P. Furtado, André L. C. Silva, Bruno A. M. Rocha

**Affiliations:** 1 Department of Biochemistry and Molecular Biology, 28121 Federal University of Ceará, Av Humberto Monte s/n, Fortaleza 60440-900, Brazil; 2 611310 Oswaldo Cruz Foundation, R. São José, S/N - Precabura, Eusébio 61760-000, Brazil; 3 TaqMol Diagnostics, 28121 Federal University of Ceará, Av Humberto Monte s/n, Fortaleza 60440-900, Brazil

## Abstract

Secretory immunoglobulin A1 (sIgA1) is the predominant antibody in mucosal secretions and human colostrum, where it plays critical roles in immune exclusion and microbiota regulation. Due to its high degree of glycosylation, oligomeric variability, and structural flexibility, selective purification of sIgA1 remains a technical challenge—yet essential for biomedical, immunological, and biotechnological applications, including diagnostic platforms and passive immunotherapy. In this study, frutalin (FTL), a galactose-specific lectin from *Artocarpus incisa*, was used as both a selective ligand and a structural probe to investigate its interaction with native sIgA1. FTL was immobilized on CNBr-activated agarose to generate an affinity matrix (frutalin–agarose), enabling the efficient purification of sIgA1 from human colostrum. The antibody was further refined by size exclusion chromatography to isolate the dimeric form, which was used for complex formation under native conditions. The resulting FTL–sIgA1 complex was characterized by dynamic light scattering (DLS), biolayer interferometry (BLI), and hemagglutination assays. DLS titration revealed cooperative multivalent binding, with a dominant population centered at ∼32 nm. BLI confirmed high-affinity interaction (*K*_D_ = 3.58 nM), modeled as a bivalent analyte system. Hemagglutination assays showed partial retention of lectin activity, indicating selective but nonsaturating engagement of carbohydrate recognition domains. Negative-staining TEM and cryo-EM provided preliminary structural evidence of complex formation. These findings highlight FTL as a stable glycan-specific ligand for mucosal antibodies and support its application in glycoprotein purification, immunoaffinity systems, and structural glycobiology.

## Graphical abstract

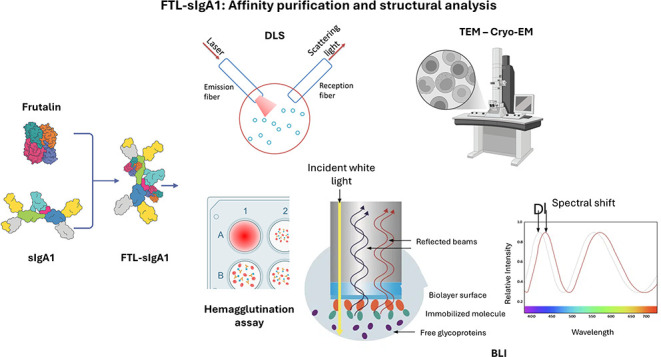

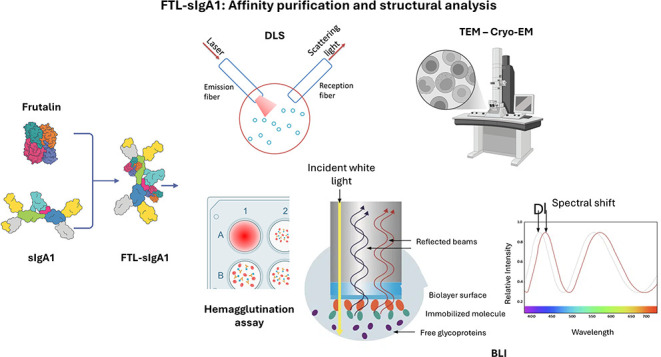

## Introduction

Lectins are a structurally diverse class of carbohydrate-binding proteins capable of recognizing specific glycan motifs without catalyzing glycosidic bond cleavage. Their unique ability to bind oligosaccharides with high selectivity and reversibility has led to broad applications in glycobiology, biotechnology, and molecular diagnostics. Among plant lectins, those from the Jacalin-related lectin (JRL) family are particularly notable for their β-prism fold and specificity for galactose- or mannose-containing glycans. These proteins often exist as stable oligomers and exhibit high functional versatility, including immunomodulatory, antiviral, antifungal, and glycoprotein-binding properties^1–3^

Frutalin (FTL), a galactose-specific JRL purified from *Artocarpus altilis* (syn. *Artocarpus incisa*) seeds (Moraceae), displays high thermal stability, well-characterized glycan recognition domains, and established multivalency.^4,5^ While previous studies have explored its diagnostic and therapeutic potentials, its capacity for selective glycoprotein capture, particularly for immunoglobulins, remains underexplored. The dense O-glycosylation of human secretory IgA1 (sIgA1), predominantly in the hinge region, renders this antibody subclass an excellent target for lectin-based recognition systems^6,7^

sIgA1 plays a crucial role in mucosal immunity and is the predominant antibody in human milk, saliva, and intestinal secretions.^8^ Structurally, it is a polymeric glycoprotein composed of two IgA1 monomers linked via a J-chain and complexed with a secretory component (SC), resulting in a highly flexible, glycosylation-rich molecule^9,10^ Despite its functional advantages, sIgA1 remains underutilized in immunotherapy and biotechnology due to technical challenges in large-scale purification. The conventional affinity supports, such as Protein A or Protein L, are ineffective.^11^ Lectins like Jacalin have been employed in this context, but their specificity and yield are often suboptimal.^11^

In this context, FTL emerges as a promising candidate for sIgA1 recognition, given its affinity for galactose-terminated glycans and structural similarity to Jacalin.^4,12^ However, there are no previous studies that have evaluated the molecular details of the FTL–sIgA1 interaction, nor its potential in glyco-affinity purification. This study addresses this gap by providing the first integrated biochemical, biophysical, and structural characterization of the FTL–sIgA1 complex. Using a combination of dynamic light scattering (DLS), biolayer interferometry (BLI), hemagglutination assays, and transmission electron microscopy (TEM and cryo-EM). Here, we demonstrate the specific, multivalent, and high-affinity interaction between FTL and sIgA1. Furthermore, we explore the implications of this interaction for selective antibody capture and functional modulation.

## Results and Discussion

### Purification and Biophysical Characterization of FTL

Breadfruit seeds yielded 90.2 ± 0.5 g of dry flour per 100 g of raw seeds. Aqueous extraction produced 40 mL of crude extract (CE*)* containing 9.0 ± 0.3 mg·mL^–^^1^ of protein (360 mg total; Table 1). Single-step galactose-affinity chromatography produced 80 mL of pooled FTL fractions at 1.0 mg·mL^–1^ (80 mg total), corresponding to a 22.2% recovery of soluble protein mass and an approximately 149-fold increase in specific hemagglutination activity (Table 1). These results confirm both the efficiency of the affinity purification step and the preservation of biological activity. Reported yields for seed-derived JRLs are typically modest (5–7 mg g^–^^1^ of flour), as described for *Treculia africana* isoforms.^13^ Conventional aqueous extraction of FTL usually yields <5 mg mL^–^^1^ of crude extract,^14^ whereas broader extraction methods may achieve >90% total recovery from protein-rich plant materials,^15,16^ though often at the expense of lectin activity. Thus, the mild aqueous approach adopted here provided efficient recovery of active FTL.

**Table 1:** Purification Yield and Hemagglutination Activity of Native FTL

sample	volume (mL)	protein (mg/mL)	total protein (mg)	recovery (%)	titer (HU)	specific activity (HU/μg)	purification fold
CE	40	9.0	360	100	2^3^	0.022	1×
FTL	80	1.0	80	22.2	2^18^	3.28	∼149×

Affinity chromatography on a d-galactose-agarose column yielded two main peaks (Figure 1A): a flowthrough (peak I) and a galactose-eluted bound fraction (peak II). SDS-PAGE (12%) of pooled bound fractions consistently displayed two major bands migrating near 15 and 12 kDa (Figure 1B). These bands correspond to α-chain glycoisoforms of frutalin; the small β-chain (∼3 kDa) is typically not visualized by SDS-PAGE. This microheterogeneity, arising from differential glycosylation and limited proteolysis, is characteristic of jacalin-related lectins^4,17,18^ and does not impair activity, as confirmed here by hemagglutination assays.

**Figure 1:**
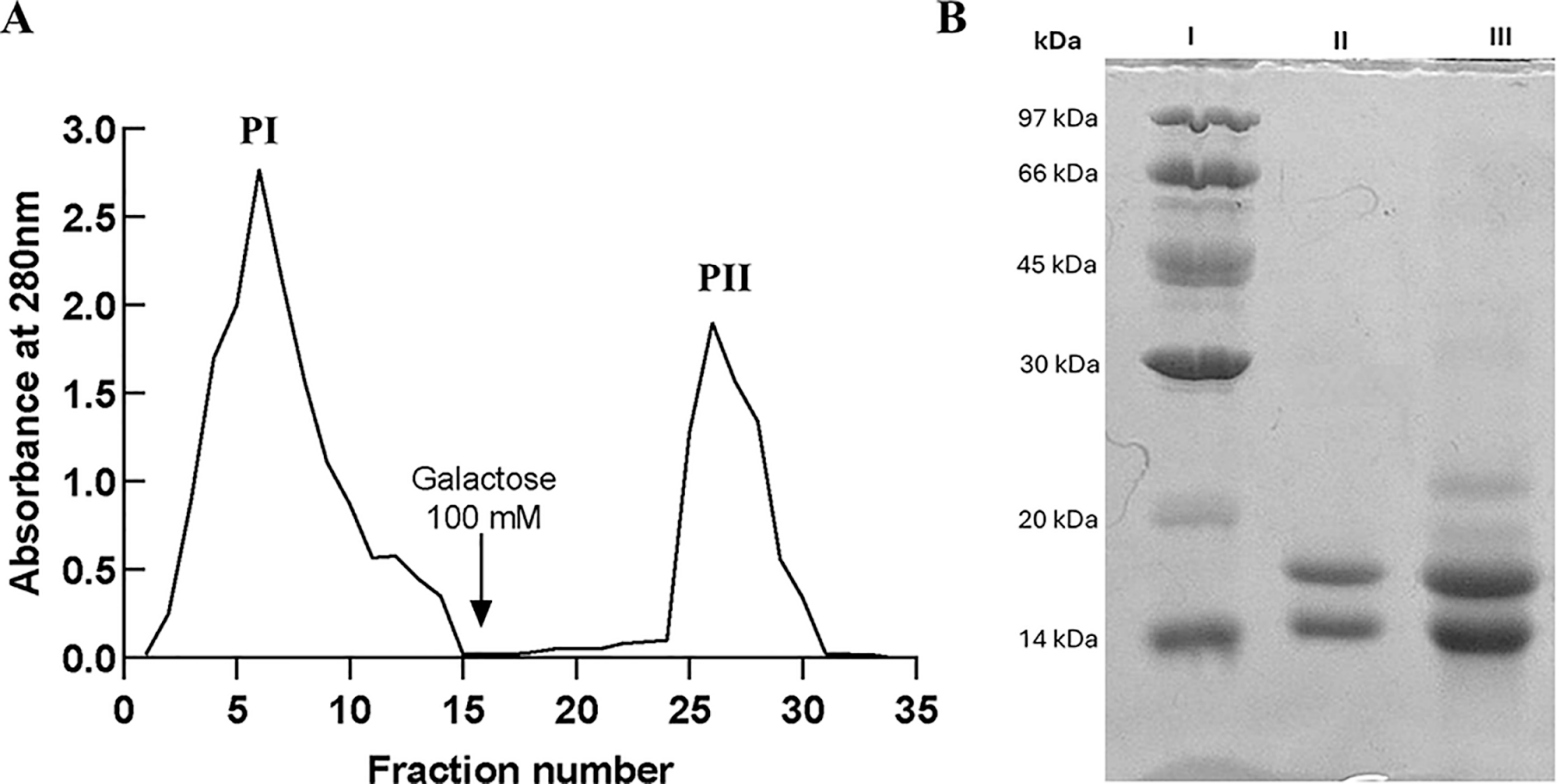
Purification of native frutalin (FTL). (A) Affinity chromatography on a d-galactose-agarose column. Peak I (PI) corresponds to unbound proteins; peak II (PII) represents FTL, eluted with 100 mM d-galactose (arrow). Absorbance was monitored at 280 nm. (B) SDS-PAGE (12%) under reducing conditions. Lane I, molecular weight marker; lane II, purified FTL (peak II). Two major bands migrating near 15 and 12 kDa are evident, consistent with the expected microheterogeneity of FTL; lane III, crude extract (CE).

DLS analysis of purified FTL revealed a *Z*-average hydrodynamic diameter (*Dz*) of 11.0 nm. The cumulant polydispersity index (PDI) was 0.387, and the coefficient of variation of the size distribution (Pd) was 48%, indicating moderate heterogeneity. Although the PDI exceeded the conventional threshold for monodisperse solutions (PDI < 0.2),^19,20^ the volume-weighted distribution showed a single dominant population centered at ∼11 nm representing >95% of the mass (Figure 2A,B). Autocorrelation functions and cumulant fits confirmed the reliability of the measurement (Figure S1A). These data indicate that purified FTL is predominantly present as a uniform oligomeric species in solution.

**Figure 2:**
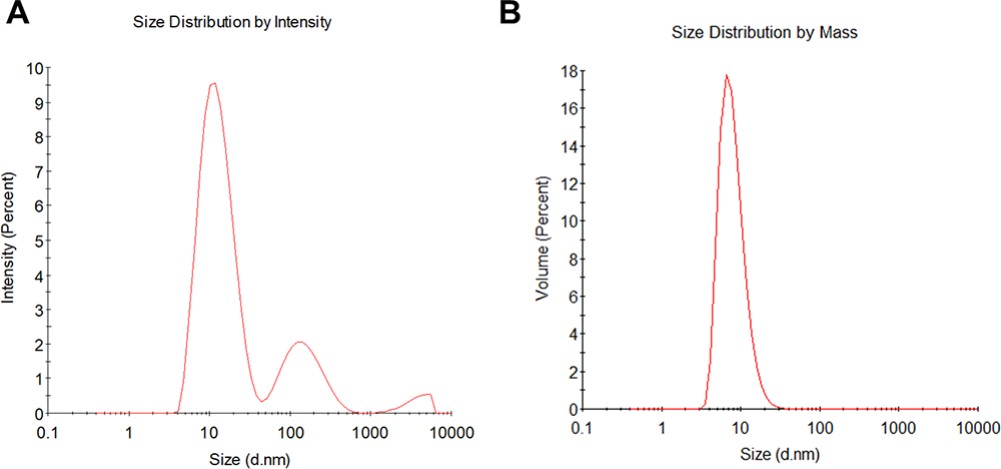
Dynamic light scattering (DLS) analysis of native frutalin (FTL). (A) Intensity-weighted size distribution showing an apparent main peak at ∼11 nm and minor signals at larger diameters (>100 nm), reflecting trace aggregates. (B) Volume-weighted distribution indicating a single predominant population at ∼11 nm, representing >95% of the total sample mass.

Size-exclusion chromatography (SEC) on a Superdex column equilibrated in PBS + 50 mM d-galactose produced a single symmetric peak at Ve = 15.57 mL (Figure S2). Using *V*_0_ = 8.96 mL and *V*_t_ = 23.562 mL, Kav = 0.453, which corresponded to an apparent molecular mass of ≈38.5 kDa based on the calibration curve (Figure S3 and Table S1). Considering the SDS-PAGE profile, this apparent mass is consistent with a dimer of αβ protomers (∼36 kDa). The hydrodynamic size determined by DLS (*Dz* = 11.0 nm) further supports this interpretation.

Previous studies reported that FTL assembles into tetramers (αβ)_4_ only under alkaline conditions (pH ≈ 10, above its isoelectric point), whereas at neutral pH, it predominantly exists as dimers.^21,22^ Our findings agree with this pH-dependent oligomerization model: Under neutral conditions, FTL remains dimeric, while tetramerization is induced only under alkaline pH. Comparable behavior is observed in other JRLs: Jacalin forms tetramers at physiological pH, AcmJRL exists as stable dimers in neutral buffers,^23^ and the rice lectin OsJAC1 oligomerizes without alkaline induction.^1,24^ Importantly, SEC and DLS provide apparent rather than absolute molecular masses, as these measurements are influenced by molecular shape, hydration, and glycosylation.^3,25,26^ Therefore, the dimeric stoichiometry proposed here should be regarded as provisional until confirmed by absolute-mass methods such as SEC-MALS, native MS, or analytical ultracentrifugation.

### Purification and Structural Characterization of sIgA1

Affinity chromatography on an FTL-conjugated agarose matrix yielded a major protein fraction from human colostrum (Figure 3A). SDS-PAGE analysis under reducing conditions confirmed the identity of sIgA1 (Figure 3B), showing the expected subunits: secretory component (∼70 kDa), heavy chains (∼50 kDa), and light chains (∼25 kDa) (Figure 3B). On average, 19.5 mg of sIgA1 was obtained from 15 mL of colostrum, corresponding to ∼10.8% recovery (Table 2). This value is consistent with the natural abundance of sIgA1 in colostrum (1–2 mg/mL, or 6–13% of total soluble protein)^27^ and demonstrates selective enrichment. Compared with immobilized jacalin, which typically yields ∼0.4 mg/mL, the FTL-based system achieved higher recovery, while also avoiding the subtype bias observed in Protein L matrices.^10,28^

**Figure 3:**
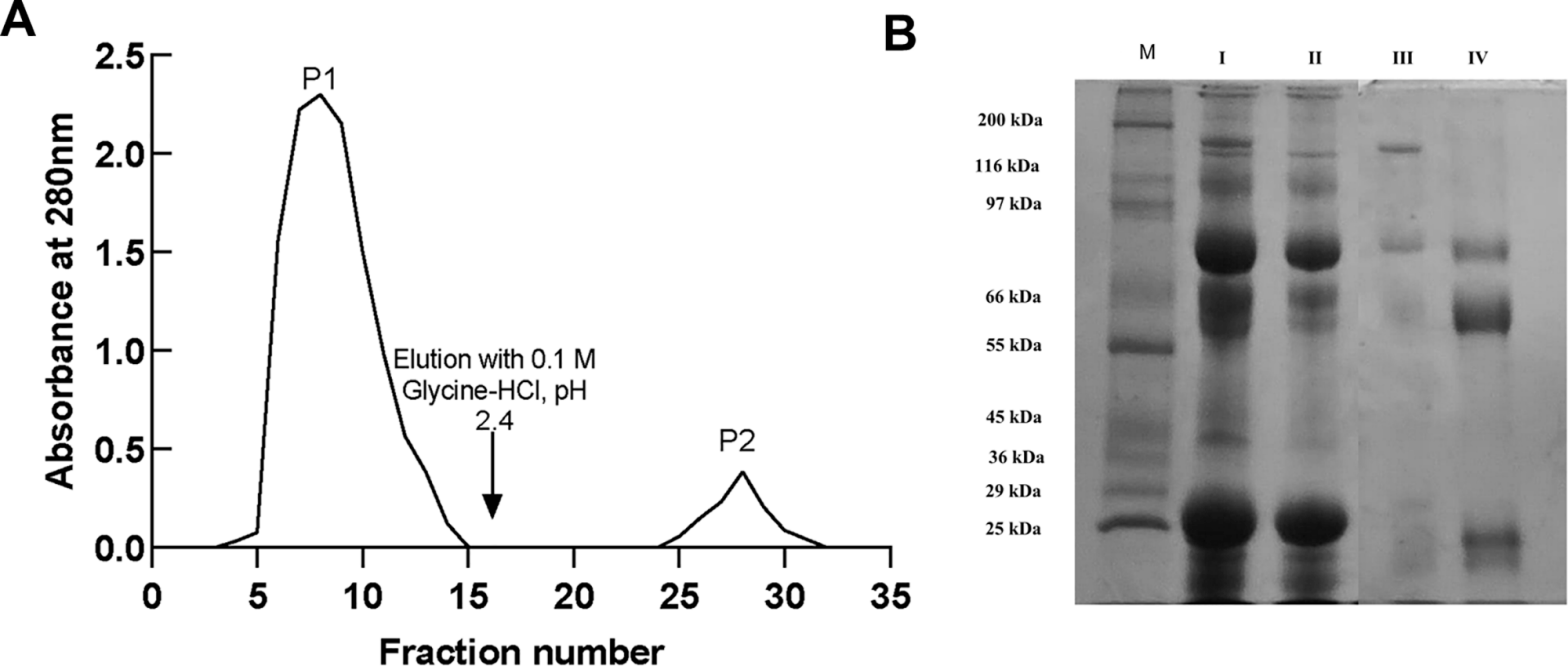
(A) Purification of sIgA1 from human colostrum by affinity chromatography on an FTL-conjugated agarose matrix. Peak I (PI) corresponds to unbound proteins; peak II (PII) represents sIgA1, eluted with 0.1 M glycine pH 2.6 (arrow). Absorbance was monitored at 280 nm. (B) SDS–PAGE (15%) under reducing conditions. Lane M, molecular weight marker; lane I, Colostrum; lane II, F060 Colostrum; lane III P1; lane IV, P2.

**Table 2:** Yield and Purity of sIgA1 Purification from Pooled Human Colostrum Using the FTL Matrix

step	volume (mL)	protein (mg/mL)	total protein (mg)	yield (%)
crude colostrum supernatant	15	12.0	180	100
sIgA1	7.5	2.6	19.5	10.8

SEC revealed multiple peaks (Figure 4A). The fraction P2 (Ve = 10.44 mL) corresponded to an apparent molecular mass of ∼316 kDa according to calibration. Although lower than the theoretical value for dimeric sIgA1 (∼400 kDa), such deviation is expected due to glycosylation and asymmetric structure.^10,27^ Earlier (P1) and later peaks (P3–P5) showed identical SDS-PAGE profiles (Figure 4B), indicating that they are sIgA1 assemblies of different oligomeric states rather than contaminants. A summary of elution volumes, Kav values, and apparent molecular masses is provided in Table S1 (Supporting Information). Fraction P2, corresponding to the dimeric population, was used for subsequent analyses.

**Figure 4:**
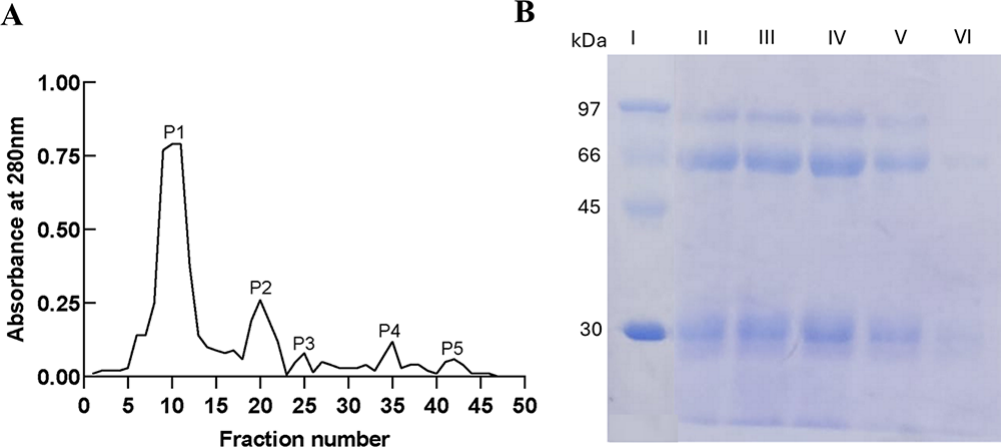
(A) Purification of sIgA1 from SEC on Superdex S-200 increase 10/300 GL. Absorbance was monitored at 280 nm. (B) SDS-PAGE (15%) under reducing conditions. Lane I: molecular weight marker; lane II: P1; lane III: P2; lane IV: P3; lane V: P4; lane VI: P5.

DLS of the P2 fraction showed a major species with *D*_*z*_ ∼ 24 nm, accounting for >95% of the sample volume (Figure 5A,B). Minor micrometer-scale aggregates were also detected but contributed negligibly to mass distribution. The high polydispersity index (PDI = 0.673) reflects the intrinsic heterogeneity of secretory IgA1, which exists as dimers, tetramers, and higher-order complexes in colostrum.^19,29,30^ These findings are consistent with reported *D*_*z*_ values for functional secretory IgA1 (18–25 nm).^7,27,31^

**Figure 5:**
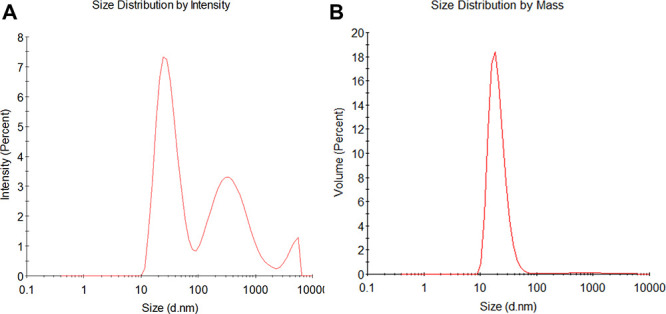
Dynamic light scattering (DLS) analysis of purified sIgA1. (A) Size distribution by intensity reveals a multimodal profile with three populations centered around ∼24 nm (major), ∼3.4 μm, and ∼5.5 μm. The intensity peak at 24 nm accounts for 97.8% of the scattering signal. (B) Volume-based distribution confirms the predominance of a single population (∼24 nm), representing 97.3% of the total sample mass.

Together, affinity purification and SEC provided structurally intact sIgA1, and DLS confirmed the predominance of a native dimeric population despite intrinsic heterogeneity.

### Formation and Characterization of the FTL–sIgA1 Complex

#### Stoichiometry and Binding Cooperativity (DLS-Based Hill Model)

DLS was used to monitor FTL–sIgA1 complex formation in solution. Incremental titration of sIgA1 with FTL (0.06–1.94 mg/mL) generated a cooperative binding curve when plotting the *Z*-average hydrodynamic diameter (*D*_*z*_) as a function of molar ratio (Figure 6A). Nonlinear fitting with the Hill equation yielded an apparent dissociation constant (*K*_D_) in the micromolar range and a Hill coefficient (*h*) > 1, indicating positive cooperativity.^32^ This suggests that engagement of one glycan site on sIgA1 by FTL enhances subsequent binding events, consistent with multivalent lectin–glycoprotein recognition.

**Figure 6:**
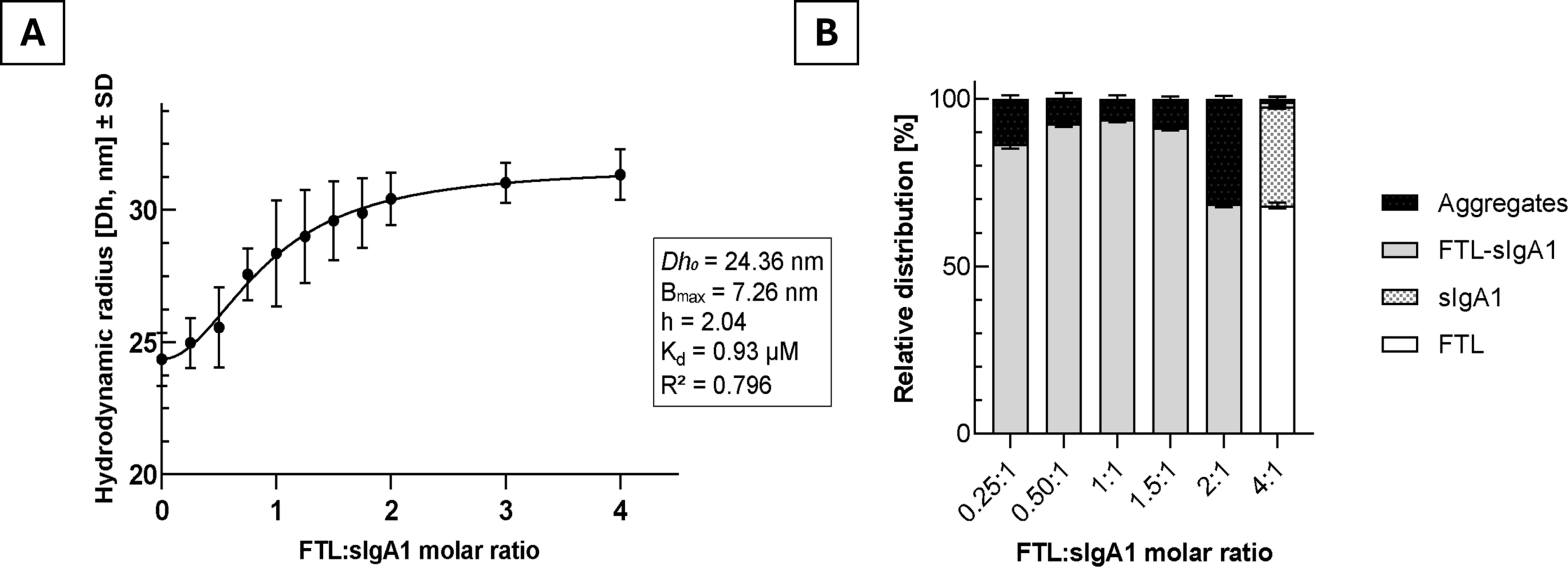
DLS-based analysis of FTL–sIgA1 complex formation. (A) Cooperative increase in *Z*-average hydrodynamic diameter (*D_z_*) with rising FTL:sIgA1 ratios, reaching a plateau near 1:1–1.5:1. The Hill fit indicated positive cooperativity (*nH* > 1) with an apparent *K*_D_ in the micromolar range. (B) Volume-based population distribution showing the predominance of the FTL–sIgA1 complex at near-equimolar ratios. Excess FTL led to the appearance of free lectin and minor aggregates, defining a saturation point.

Comparable Hill coefficients have been reported for other multivalent lectin–glycan systems, including DC-SIGN (*Dendritic Cell-Specific Intercellular adhesion molecule-3-Grabbing Nonintegrin*) binding to glycosylated nanoparticles and jacalin interactions with IgA fragments, where *h* > 1 reflects enhanced avidity through multiple simultaneous contacts.^33,34^ Such cooperativity is expected for FTL, given its multiple CRDs and the dense glycosylation of sIgA1. Importantly, the Hill model here provides a functional description of cooperative binding in solution, rather than mechanistic or kinetic detail.^32,35^

The binding curve reached a plateau at an ∼1:1 to 1.5:1 molar ratio (FTL:sIgA1), with a stable *D*_*z*_ of ∼27 nm, consistent with the formation of a high-molecular-weight complex. Volume-based analysis (Figure 6B) confirmed this predominant population, while higher lectin ratios produced additional peaks from free FTL and minor aggregates, indicating a clear saturation point. Although the PDI of the complex was moderately high (∼0.5), such heterogeneity is inherent to supramolecular assemblies involving antibodies, as reported for IgA-containing immune complexes and lectin-based glycoprotein networks.^31,36^ The predominance of a single major species in the volume distribution demonstrates that heterogeneity did not compromise the main binding event.

From a functional perspective, defining the optimal stoichiometry and demonstrating positive cooperativity provides a framework for applied systems. Identifying the saturation point prevents efficiency loss due to excess lectin and highlights the potential of FTL as a multivalent recognition module for affinity matrices and biosensors. To our knowledge, this is the first quantitative demonstration of positive cooperativity in a lectin–IgA system using DLS and Hill analysis.

To further assess this behavior, size-exclusion chromatography (SEC) was performed under competitive conditions with 50 mM galactose in the mobile phase (Figure S4). In this setting, the complex largely dissociated, producing a peak at Ve = 15.4 mL corresponding to free FTL (∼39 kDa apparent) and a peak at Ve = 9.4 mL (∼488 kDa apparent), consistent with residual high-avidity complexes. For comparison, sIgA1 alone analyzed by SEC (without galactose) eluted at Ve = 10.44 mL (∼318 kDa apparent), in agreement with its dimeric form. These findings confirm that the FTL–sIgA1 interaction is carbohydrate-dependent, reversible, and multivalent. The persistence of a small high-mass fraction under competitive conditions suggests that some complexes achieve strong avidity and resist complete dissociation.

Taken together, DLS and SEC provide complementary evidence: DLS reveals cooperative supramolecular assembly with an apparent *K*_D_ and saturation stoichiometry, while SEC demonstrates the reversibility and carbohydrate dependence of interaction. Despite limitations in deriving absolute molecular masses from either technique—SEC due to glycosylation/asymmetry and DLS due to polydispersity—the convergence of these data strongly supports the conclusion that FTL forms stable multivalent complexes with sIgA1 through glycan-mediated binding.

#### Kinetic Analysis by Biolayer Interferometry

The interaction between FTL and sIgA1 was further characterized by biolayer interferometry. Sensorgrams displayed concentration-dependent binding with rapid association and partially reversible dissociation (Figure 7A). Global fitting was best described by a bivalent analyte model (1:2, ligand:analyte), consistent with the structural multivalency of both molecules. This model assumes that one immobilized sIgA1 molecule engages two FTL molecules via distinct glycan epitopes, reflecting cooperative binding behavior.

**Figure 7:**
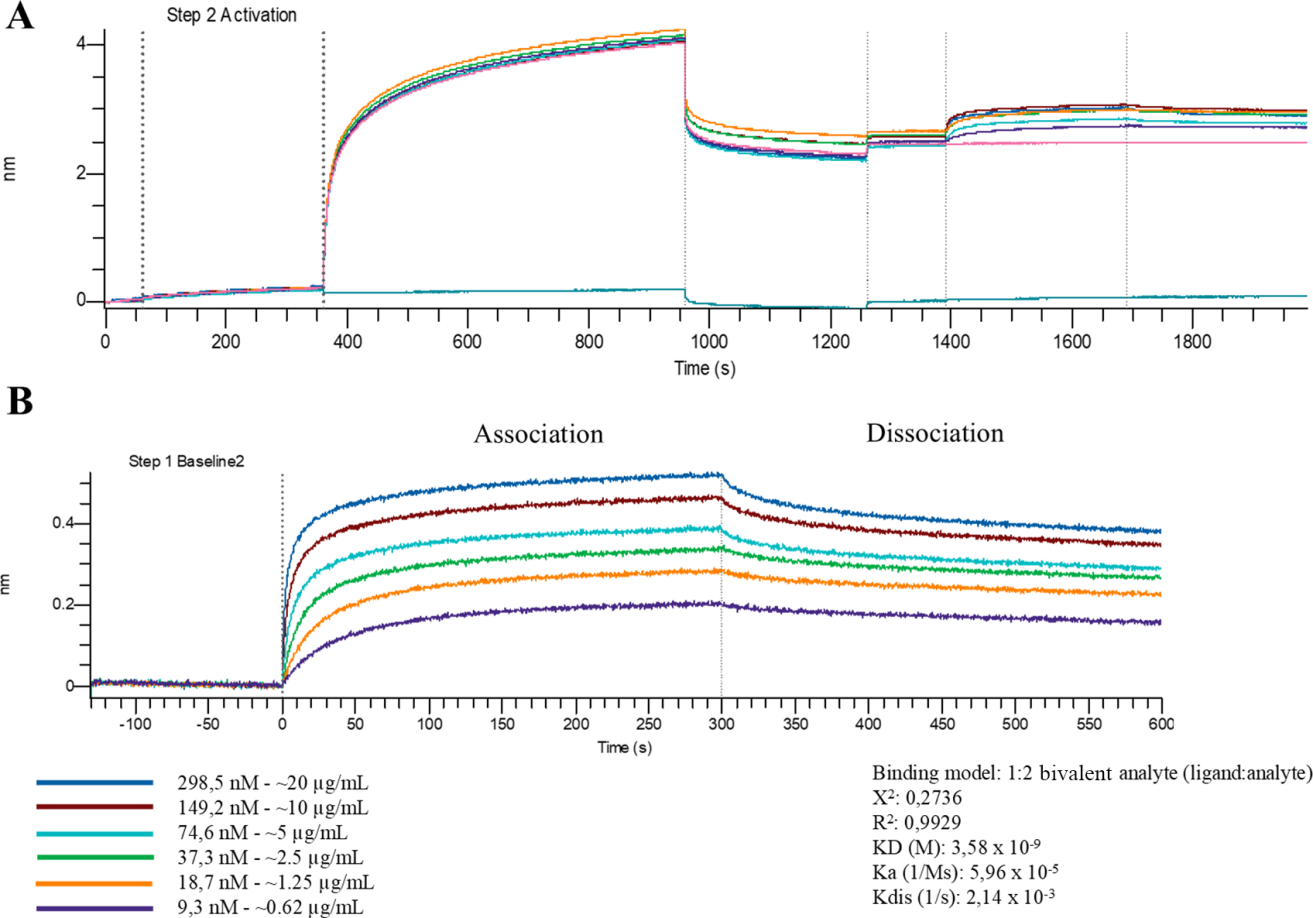
Characterization of sIgA1-FTL binding kinetics using biolayer interferometry (BLI). Representative sensorgrams showing the association and dissociation of FTL analytes at increasing concentrations (9.3 nM to 298.5 nM) to immobilized sIgA1. (A) Overall binding profile including a secondary activation step. (B) Zoomed kinetic curves with calculated binding parameters. Analysis using a 1:2 bivalent analyte (ligand analyte) model provided a dissociation constant (KD) of 3.58 × 10^–9^ M, an association rate constant (*K*_a_) of 5.96 × 10^–5^ 1/Ms, and a dissociation rate constant (*K*_dis_) of 2.14 × 10^–3^ 1/s.

The fitted kinetic parameters revealed a high-affinity interaction, with *K*_D_ = 3.58 nM, *K*_a_ = 5.96 × 10^5^ M^–^^1^ s^–^^1^, and *K*_dis_ = 2.14 × 10^–^^3^ s^–^^1^ (Table 3 and Figure 7B). The negative free energy of binding (Δ*G*° = −48.8 kJ·mol^–^^1^) confirmed that complex formation is spontaneous and thermodynamically favorable. The excellent fit quality (*R* ^2^ = 0.993; χ^2^ = 0.274) further validated the robustness of the analysis.

**Table 3:** Kinetic Parameters and Model Fitting for the sIgA1-FTL Interaction^a^

parameter	score	unit	interpretation
*K*_D_	3.58 × 10^–^^9^	mol·L^–^^1^ (M)	high affinity
*K*_a_	5.96 × 10^5^	1·mol^–^^1^·s^–^^1^	quick association
*K*_dis_	2.14 × 10^–^^3^	s^–^^1^	slow and steady dissociation
Δ*G*°	–48.8	kJ·mol^–^^1^	spontaneous and highly supportive
Modelo	1:2		compatible with multivalence of both molecules
*R* ^2^	0.9929		excellent
χ^2^	0.2736		low model error

a The table summarizes the binding affinity and kinetic rates obtained from BLI analysis, along with interpretation of each parameter. The data were fitted using a 1:2 bivalent analyte (ligand:analyte) model, indicating multivalent interactions between sIgA1 and FTL.

These findings complement the DLS-based Hill analysis, which estimated an apparent *K*_D_ in the micromolar range and revealed positive cooperativity in solution. The apparent discrepancy in affinity values arises from the different principles of the techniques: DLS reports a functional binding parameter derived from hydrodynamic size transitions in supramolecular assemblies, whereas BLI directly measures kinetic association and dissociation at the molecular interface.^36–38^ Both approaches converge in demonstrating a strong, multivalent, and cooperative interaction between FTL and sIgA1.

The use of BLI in lectin–glycoprotein systems has been extensively validated in the literature, particularly in multivalent contexts where classical models fail to describe the kinetics accurately.^7,37–41^ The successful modeling of the FTL–sIgA1 interaction supports the use of BLI as a sensitive platform for functional characterization and process development. These findings also reinforce the potential of FTL as a biotechnological tool for glycoprotein capture, contributing to the design of affinity matrices for preparative sIgA1 purification.

Together with SEC and DLS results, the BLI data establishes that FTL recognizes sIgA1 with nanomolar affinity through a multivalent mechanism. This dual-level characterization—functional in solution and kinetic at the interface—provides a robust foundation for the application of FTL as a glycan-binding module in biotechnological platforms, including preparative sIgA1 purification and biosensing.

#### Functional Implications and Hemagglutination Inhibition

The functional consequences of complex formation between FTL and sIgA1 were assessed using a hemagglutination assay. Samples of FTL and the preformed FTL–sIgA1 complex (all at 1 mg/mL) were tested in 96-well microplates. Free FTL exhibited a high agglutination titer (∼2^17^), reflecting its strong affinity for galactose residues on the erythrocyte surface. As expected, sIgA1 alone showed negligible activity (∼2^4^). The FTL–sIgA1 complex maintained a relatively high titer (∼2^16^), but this value was significantly reduced compared to free FTL (ANOVA, *p* < 0.05).

This moderate reduction suggests partial masking of FTL’s carbohydrate recognition domains (CRDs) upon complex formation, possibly due to steric hindrance or glycan–glycan interactions.^42^ To further explore this effect, competitive inhibition assays were performed using d-galactose (50, 100, and 200 mM). Both FTL and the FTL–sIgA1 complex showed dose-dependent inhibition, with titers dropping to ∼2^9^ and ∼2^8^, respectively, at 200 mM galactose. These findings indicate that despite complex formation, CRDs remain accessible to free galactose, confirming the reversibility and specificity of the interaction.^43^

The observed behavior is consistent with reports for other oligomeric lectins, such as cMoL from *Moringa oleifera*, which functional activity is preserved through multivalent binding despite conformational changes.^44^ Quantitative analysis of relative titer reduction (Δlog_2_) across galactose concentrations further confirmed that complexation subtly modulates FTL’s hemagglutination capacity (Figure 8, *p* < 0.05).

**Figure 8:**
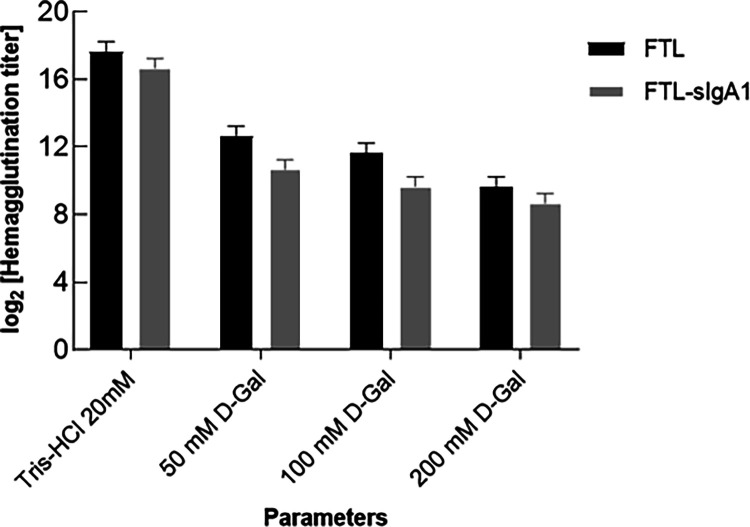
Hemagglutination assay of FTL and FTL-sIgA1 complex. Log_2_ hemagglutination titers are shown for FTL (black bars) and the FTL-sIgA1 complex (gray bars) under different conditions, including Tris–HCl 20 mM and varying concentrations of d-galactose. Error bars represent standard deviation.

Together with DLS and BLI data, these results support a model in which FTL binds sIgA1 through specific and multivalent glycan-mediated interactions nanomolar-range affinity. The complex remains functionally competent, with partial preservation of hemagglutination activity. The decrease in activity upon complexation highlights the role of sIgA1 glycans in engaging FTL binding sites, without fully abrogating lectin function.

#### Electron Microscopy Analysis of the FTL–sIgA1

Preliminary structural characterization was performed by transmission and cryo-electron microscopy. Preliminary structural evaluation of the FTL–sIgA1 complex was performed using transmission electron microscopy with negative staining (TEM) and cryo-electronic microscopy (cryo-EM). These analyses aimed to assess particle morphology, size distribution, and the suitability of samples for high-resolution structural studies.

##### TEM Analysis

Negative-staining TEM micrographs were acquired at 80 kV using protein samples at 0.05 mg·mL^–^^1^. The SEC-purified sIgA1 P2 fraction showed dispersed, well-defined particles with characteristic “Y”-shaped morphology and an average diameter of ∼18 nm (Figure 9A). FTL appeared as globular particles of ∼9 nm with uniform distribution (Figure 9B), consistent with its oligomeric form previously described for JRL.^4,17,45^ The P1-SEC fraction of sIgA1, corresponding to higher-order oligomers or aggregates, displayed increased heterogeneity and particle size (∼28 nm), while maintaining structural integrity (Figure 9C). The FTL–sIgA1 complex, derived from the SEC-purified dimeric fraction, presented as heterogeneous particles averaging ∼22 nm (Figure 10), consistent with stable multivalent assemblies between lectins and glycoproteins.^45^

**Figure 9:**
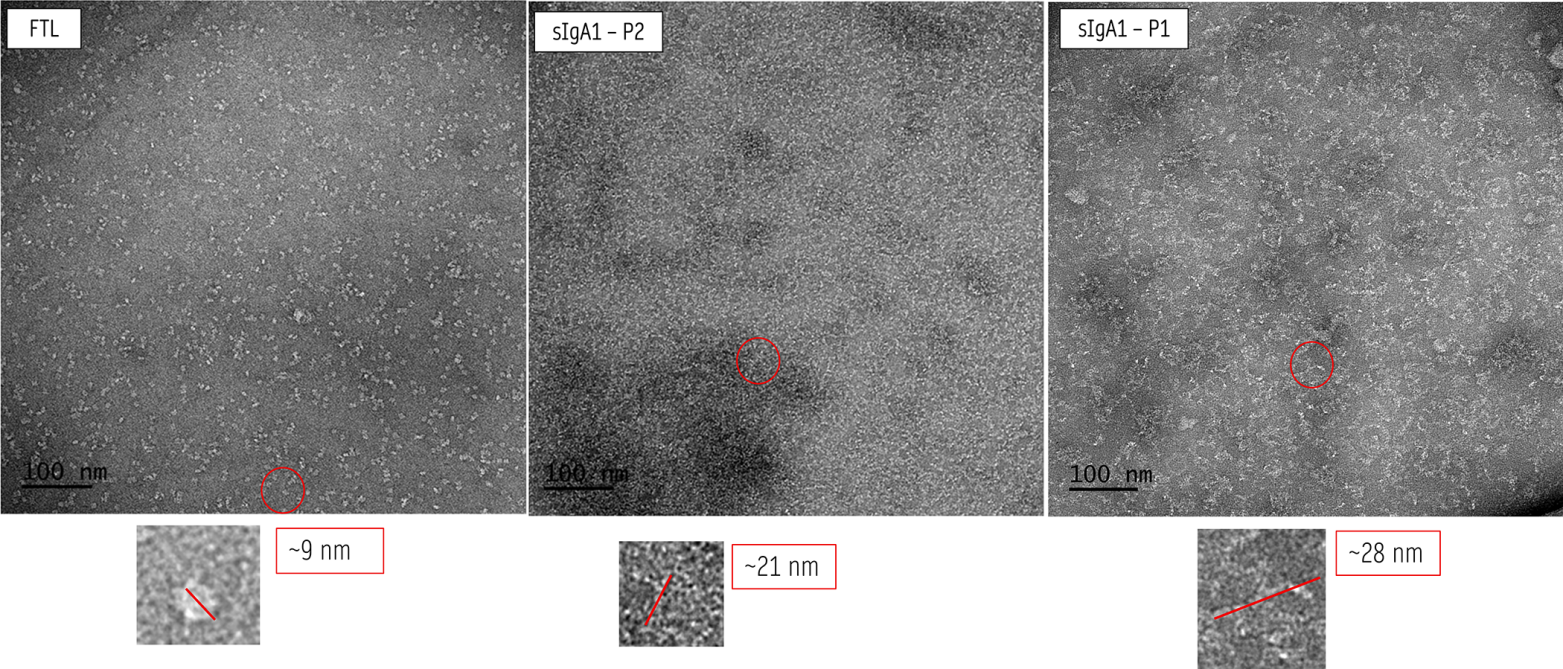
Negative staining TEM analysis of purified sIgA1. (A) FTL particles (∼9 nm) displaying globular morphology and uniform distribution (B) SEC-purified sIgA1 P2 fraction, showing well-defined “Y”-shaped particles with an average diameter of ∼18 nm FTL particles (∼9 nm) displaying globular morphology and uniform distribution. (C) sIgA1 P1 fraction, enriched in higher-order oligomers, exhibiting heterogeneous particles with an average diameter of ∼28 nm.

**Figure 10:**
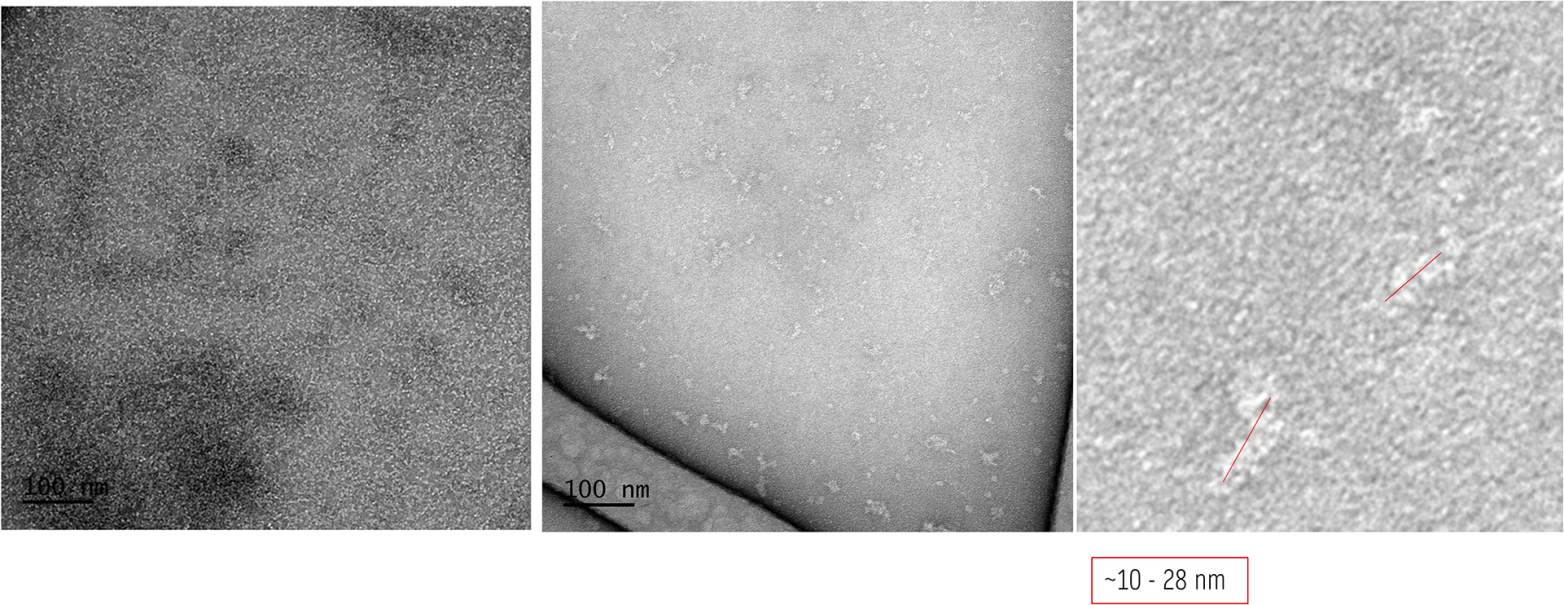
Negative-staining TEM analysis of the FTL–sIgA1 complex. SEC-purified dimeric sIgA1 (P2) was incubated with native FTL, resulting in heterogeneous particles averaging ∼22 nm. Morphology indicates stable supramolecular assemblies consistent with multivalent lectin–glycoprotein interactions.

These morphological observations are in agreement with previously reported dimensions for polymeric IgA and related lectins,^46,47^ and support the DLS results, where a predominant population of ∼24–27 nm was observed for the complex. The slight differences between DLS and TEM values are expected, since DLS measures the hydrodynamic diameter in solution, whereas TEM provides the projected molecular size under staining and drying conditions. Comparative cryo-EM and DLS studies of sIgA1 have reported similar ranges of 18–30 nm and conformational variability due to glycosylation and multimeric states, further validating the present findings.^46,47^ Together, these results confirm that FTL and sIgA1 form stable supramolecular assemblies that can be directly visualized by TEM, supporting the biophysical evidence from SEC and DLS.

##### Cryo-EM Screening

Vitrified samples of FTL, sIgA1, and the FTL–sIgA1 complex were imaged using a Talos Artica G2 microscope (200 kV) under controlled conditions. Grids were prepared with the Vitrobot Mark IV using a blot force of 2, blot time of 4 s, and wait time of 5 s. FTL samples at 0.1 mg mL^–1^ formed thin, uniform ice with good particle distribution (Figure 11A). In contrast, sIgA1 samples exhibited vitrification artifacts, including heterogeneous ice and “leopard skin” texture associated with aggregation (Figure 11B). These issues are common for large, flexible glycoproteins and reflect sIgA1’s structural complexity.^48,49^

**Figure 11:**
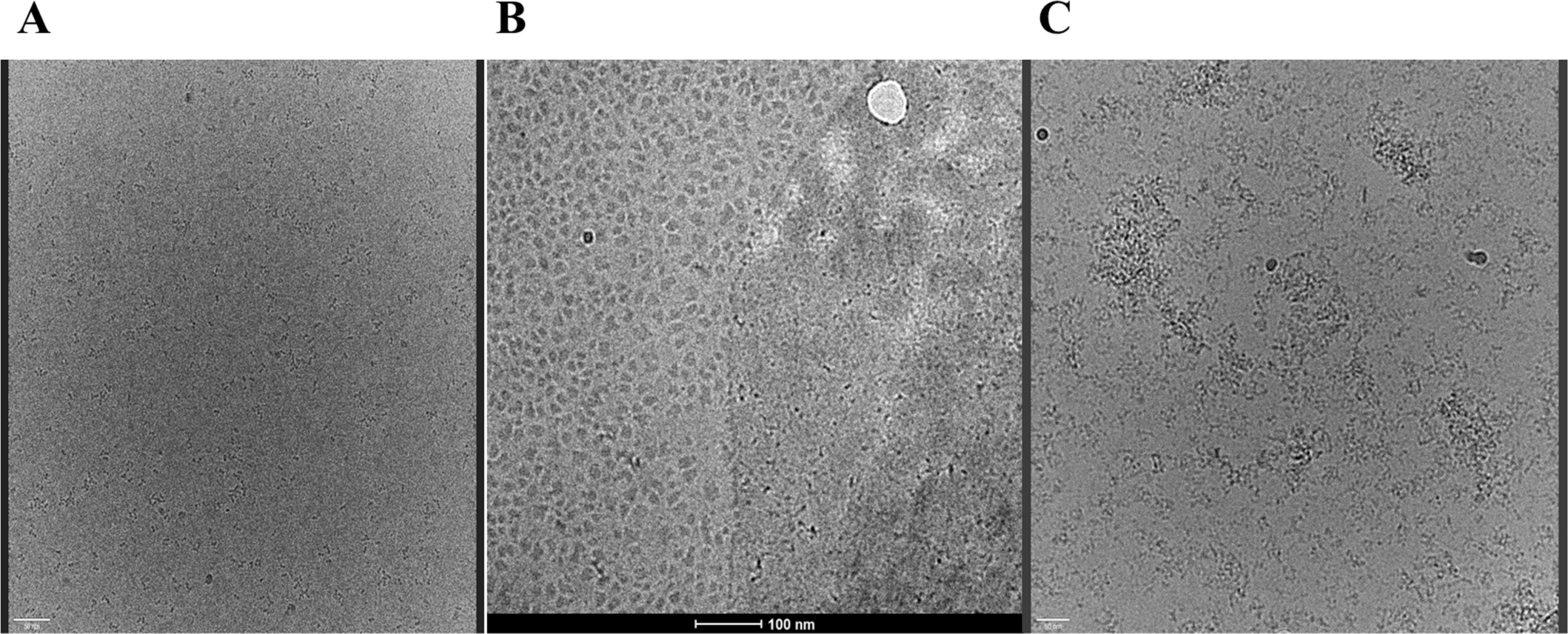
Representative micrographs obtained after vitrification of samples on Quantifoil R2/2 grids (300 mesh Cu), prepared on the Vitrobot with blotting force of −2, blotting time of 4 s, and waiting time of 5 s. (A) Isolated FTL sample (0.1 mg/mL), showing amorphous and thin ice, with good uniformity and no visible agglomeration. (B) Isolated sIgA1 (0.1 mg/mL), exhibiting a typical “leopard skin” pattern due to the formation of heterogeneous ice and peripheral accumulation of particles, associated with the conformational flexibility of the molecule. (C) FTL–sIgA1 complex (2:1 ratio, 0.25 mg/mL), with partial formation of thin ice in some regions and the presence of agglomerates distributed throughout the field, suggesting multivalent interactions and a tendency to aggregation.

The vitrified complex at 0.25 mg/mL (2:1 FTL:sIgA1 ratio) displayed localized thin ice regions but also showed particle clustering and preferential adsorption to grid edges (Figure 11C). These patterns suggest intrinsic multivalency-induced aggregation and sensitivity to air–water interface effects, as described in cryo-EM literature for similar macromolecular assemblies.^50–52^

Despite the structural insights obtained, we recognize that our cryo-EM data sets were limited by issues common to glycoprotein–lectin assemblies, such as adsorption to the air–water interface, particle heterogeneity, and variable ice thickness.^52,53^ These constraints precluded achieving near-atomic resolution in the present study. Recent advances in sample-preparation strategies, including the use of continuous carbon films, affinity grids with random biotinylation, low-percentage protective additives, and self-wicking deposition devices, have been shown to mitigate such challenges and improve particle distribution.^48,49,53^ Future work implementing these approaches will likely overcome the current limitations, enabling high-resolution structural elucidation of the FTL–sIgA1 complex.

## Conclusions

The present work presents a comprehensive biochemical and biophysical characterization of the interaction between frutalin (FTL), a galactose-binding plant lectin, and secretory immunoglobulin A1 (sIgA1), the major antibody in human colostrum. FTL was successfully employed as an affinity ligand to purify native sIgA1 with high selectivity and yield. The purified proteins were then used to form a stable complex, which was analyzed by DLS, BLI, hemagglutination, and electron microscopy.

Biophysical assays revealed multivalent and cooperative binding between FTL and sIgA1, with nanomolar-range affinity and preservation of lectin functionality upon complexation. Structural screening by TEM and cryo-EM provided preliminary morphological data, supporting the formation of supramolecular assemblies and informing conditions for future high-resolution studies.

Future work might focus on improving vitrification protocols and exploring cryo-EM-compatible formulations to enable structural resolution of the FTL–sIgA1 complex at the atomic level. Additionally, the successful use of frutalin as a selective ligand reinforces its potential for the development of scalable affinity matrices, biosensors, and targeted delivery systems involving glycosylated biomolecules.

Together, these findings expand the understanding of lectin–antibody interactions and pave the way for novel applications in immunotechnology, glycoprotein purification, and structural glycobiology.

## Experimental Section

### Materials

Galactose-agarose resin was purchased from Sigma-Aldrich (St. Louis, Missouri, USA). Human colostrum samples were collected from voluntary donors under approved ethical protocols (CAAE 09043512.0.0000.5050). All reagents were of analytical grade and obtained from standard commercial suppliers. DLS was performed using a Zetasizer Nano ZS (Malvern Instruments), BLI on an Octet RED96 (Sartorius), TEM on a JEOL JEM-1400, and cryo-EM on a Thermo Fisher Scientific Talos Arctica.

### Lectin Purification

FTL was extracted from *Artocarpus altilis* seeds using PBS, 1:10 (20 mM phosphate buffer, 150 mM NaCl, pH 7.4), followed by centrifugation and clarification. The supernatant was applied to a galactose-agarose column pre-equilibrated with PBS. After extensive washing, bound lectin was eluted with 100 mM d-galactose in PBS. The eluted fractions were monitored by absorbance at 280 nm and analyzed by SDS-PAGE.

### Isolation and Purification of Human sIgA1

#### Human Colostrum Collection

Colostrum was collected within 48 h postpartum from healthy volunteers at the Maternidade Escola (UFC), under ethical approval. Samples were centrifuged at 15,000 rpm for 15 min at 4 °C. The intermediate (serum) phase was filtered (0.22 μm), aliquoted, and stored at −80 °C. Colostrum serum was precipitated with ammonium sulfate (60% saturation), centrifuged (13,000 rpm, 15 min, 4 °C), resuspended in PBS (pH 7.4), and dialyzed.

#### FTL Immobilization

FTL (4 mg/mL) was immobilized on CNBr-activated Sepharose 4B (10 mL) at 4 °C. Remaining reactive groups were blocked with 50 mM ethanolamine (pH 8.0). Immobilization efficiency was assessed by absorbance at 280 nm. The matrix was packed into BioRad columns, equilibrated with PBS, and stored at 4 °C.^14^

#### Size-Exclusion Chromatography (SEC) and Calibration

SEC was carried out on a Superdex 200 Increase 10/300 GL column (Cytiva) using an ÄKTA Pure system with 20 mM Tris–HCl, 150 mM NaCl (pH 7.4) as running buffer at 0.5 mL/min. Samples (200 μL, 2.0 mg/mL) were injected and elution was monitored at 280 nm. The SEC column was calibrated using purified protein standards: thyroglobulin (660 kDa), apoferritin (440 kDa), alcohol dehydrogenase (150 kDa), conalbumin (75 kDa), ovalbumin (44 kDa), carbonic anhydrase (29 kDa), ribonuclease A (13.7 kDa), and aprotinin (6.5 kDa). *K*_av_ values were calculated as (*V*_e_ – *V*_o_)/(*V*_c_ – *V*_o_), and a standard curve of log­(*M*_r_) versus *K*_av_ was generated (R^2^ = 0.992). Apparent molecular masses of FTL, sIgA1, and the FTL–sIgA1 complex were interpolated from this curve.

#### Affinity Purification of sIgA1

Colostrum proteins were applied to this FTL matrix, washed with PBS, and eluted with 0.1 M glycine (pH 2.6). Eluates were pooled, dialyzed, and concentrated.The enriched fraction (FrsIgA1) was quantified (A_2__8__0_) and analyzed by SDS-PAGE (15%). FrsIgA1 was further purified by SEC (Superdex 200 Increase 10/300 GL, Cytiva) on an ÄKTA Pure system, using 20 mM Tris–HCl, 150 mM NaCl (pH 7.4), as running buffer. A 200 μL sample (2.0 mg/mL) was injected, and elution was carried out at 0.5 mL min^–1^; fractions corresponding to dimeric sIgA1 were pooled and stored at 4 °C.

#### DLS and Hill Analysis

Dynamic light scattering was used to measure the hydrodynamic diameter (*D_z_*) of proteins in solutions, providing information on particle size and distribution. DLS detects fluctuations in scattered light caused by the Brownian motion of molecules, which are mathematically related to their apparent size in solution.

To evaluate complex formation, sIgA1 was titrated with increasing concentrations of FTL (0.06–1.94 mg/mL). The resulting *D_z_* values, which increase when molecules associate into larger assemblies, were plotted as a function of the FTL:sIgA1 molar ratio.

The binding curve was analyzed using the Hill equation, a model commonly applied to multivalent interactions:

$$Y=\frac{Y_{\max*}{\left[L\right]}^{h}}{K_{D}^{h}+{\left[L\right]}^{h}}$$

where *y* is the normalized response (relative increase in *D_Z_*), [*L*] is the concentration (or molar ratio) of FTL, *K*_D_ is the apparent dissociation constant, and *h* is the Hill coefficient indicating binding cooperativity. Nonlinear regression was performed with GraphPad Prism 8 to obtain *K*_D_ and *h*. These parameters provide a functional description of how FTL interacts with sIgA1 in solution: *K*_D_ indicates the apparent affinity of the system, while *h* reveals whether multivalent binding enhances complex formation.

#### Biolayer Interferometry

Kinetic analyses were performed using the Octet RED96 system. sIgA1 was immobilized on AR2G biosensors via amine coupling, and FTL was used as an analyte at concentrations ranging from 0.7 to 30 nM. Sensorgrams were recorded at 25 °C in PBS with 0.02% Tween-20. Binding models were fitted using Octet Data Analysis software (Sartorius), applying both monovalent and bivalent analyte models. Thermodynamic parameters were calculated from the kinetic constants using the equation Δ*G*° = −*RT* ln *KD*, where *R* = 8.314 J·mol^–^^1^·K^–^^1^ and *T* = 298 K (25 °C). Values were obtained from global fits in the Octet Data Analysis software.

#### Hemagglutination Assay

Hemagglutination assays were performed to evaluate the biological activity of native FTL (FTL), purified sIgA1, and the FTL–sIgA1 complex, following the method of Moreira and Perrone (1977). Briefly, 50 μL of PBS was mixed with 50 μL of each protein solution (1 mg/mL), and 2-fold serial dilutions were prepared in 96-well microplates. To each dilution, 50 μL of a 3% (v/v) suspension of rabbit erythrocytes was added. Plates were incubated at 37 °C for 30 min, followed by an additional 30 min at room temperature. Hemagglutination was assessed visually, and the titer was defined as the reciprocal of the highest dilution showing visible agglutination (expressed as hemagglutination units, H.U.).

For inhibition assays, the hemagglutination protocol was repeated in the presence of d-galactose at final concentrations of 50, 100, and 200 mM. Each sugar solution was prepared in PBS and mixed with the protein sample (FTL or FTL–sIgA1) before serial dilution. The mixtures were incubated for 15 min at room temperature before the addition of the erythrocyte suspension. Hemagglutination titers were compared to controls without inhibitor, and results were expressed as Δlog_2_ relative to the untreated condition.

#### Electron Microscopy

Preliminary structural analysis of the FTL–sIgA1 complex was performed using negative-staining transmission electron microscopy (TEM) and cryo-electron microscopy (cryo-EM) to evaluate particle morphology, sample homogeneity, and suitability for high-resolution imaging. All procedures were conducted at the National Nanotechnology Laboratory (LNNano) of the Brazilian Center for Research in Energy and Materials (CNPEM, Campinas, SP), with technical support from the local team.

Negative-staining TEM was performed on a JEM-1400 Plus microscope (JEOL) operating at 80 and 120 kV. A 3 μL aliquot of each sample was applied to glow-discharged carbon-coated copper grids (Quantifoil R2/2, 300 mesh), adsorbed for 1 min, stained with 2% uranyl acetate for 30 s, and blotted with filter paper. Images were acquired at 80,000× and 100,000× magnification to assess particle distribution and integrity.

Cryo-EM analysis was carried out after automated vitrification using a Vitrobot Mark IV. Samples (0.1–1.0 mg/mL) in 20 mM Tris–HCl and 150 mM NaCl (pH 7.4) were applied to glow-discharged Quantifoil R2/2 grids, blotted for 2–4 s, and plunge-frozen in liquid ethane (−168 °C) using a GridBox R2X2 system.

Vitrified grids were screened on a Talos Artica G2 microscope (Thermo Fisher, 200 kV) to assess ice quality and particle dispersion. Cassettes were clipped, assembled, and mapped for foil integrity and ice uniformity.

#### Statistical Analysis

All experiments were performed in triplicate, and results are presented as mean ± standard deviation (SD). Statistical analyses were conducted in GraphPad Prism 8. Hemagglutination assays were compared using one-way analysis of variance (ANOVA) followed by Tukey’s post hoc test, with significance defined as *p* < 0.05.

For DLS-based Hill analysis, nonlinear regression was applied to plot the hydrodynamic diameter (*D_z_*) as a function of the FTL:sIgA1 molar ratio. The fitted Hill model was statistically validated by ANOVA, comparing the Hill fit against a null (constant mean) or noncooperative (Langmuir, *h* = 1) model. A significant improvement (*p* < 0.05) was considered evidence of cooperativity. Goodness of fit was further assessed by *R* ^2^, residuals, and parameter standard errors.

## Supplementary Material



## Notes

Full dynamic light scattering (DLS) results used to characterize the formation of the FTL–sIgA1 complex; hydrodynamic behavior of the system under varying molar ratios, supporting the cooperative nature of the interaction through Hill model fitting and contributing to the robustness of the stoichiometric and conformational analysis presented in the main text (PDF)

## Abbreviations

ANOVA: analysis of variance
BLI: biolayer interferometry
CRD: carbohydrate recognition domain
DLS: dynamic light scattering
FTL: frutalin
HU: hemagglutination unit
MAC: matrix affinity chromatography
PBS: phosphate-buffered saline
PDI: polydispersity index
Rh: hydrodynamic radius
SEC: size-exclusion chromatography
SDS-PAGE: sodium dodecyl sulfate polyacrylamide gel electrophoresis
sIgA1: secretory immunoglobulin A1
TEM: transmission electron microscopy.
