# Supplementary material for: Frutalin as an Affinity Tool for sIgA1: Biophysical and Structural Characterization of the Lectin–Antibody Complex
Source: ACS Omega. 2025 Dec 5;10(49):60298–308. doi: 10.1021/acsomega.5c06812 (PMC12713436; doi:10.1021/acsomega.5c06812)
Supplement: Supplementary file 1 [file ao5c06812_si_001.pdf]

Frutalin as an Affinity Tool for sIgA1: Biophysical and Structural Characterization of the Lectin–Antibody Complex.

Roberta C. C. Costa[1], Talita A. Leite[1], José G. S. Gomes[1], Francisco P. F. Silva[1] Marcus R. L. Bezerra[2], Wallady S Barroso[3], Gilvan P. Furtado[2], André L. C. Silva[1] and Bruno A. M. Rocha*[1]

[1]Department of Biochemistry and Molecular Biology, Federal University of Ceará, Av Humberto Monte s/n, Fortaleza, 60440-900, Brazil;

[2]Oswaldo Cruz Foundation, R. São José, S/N - Precabura, Eusébio, 61760-000, Brazil;

[3]TaqMol Diagnostics, Federal University of Ceará, Av Humberto Monte s/n, Fortaleza, 60440-900, Brazil.

Supplementary Figures

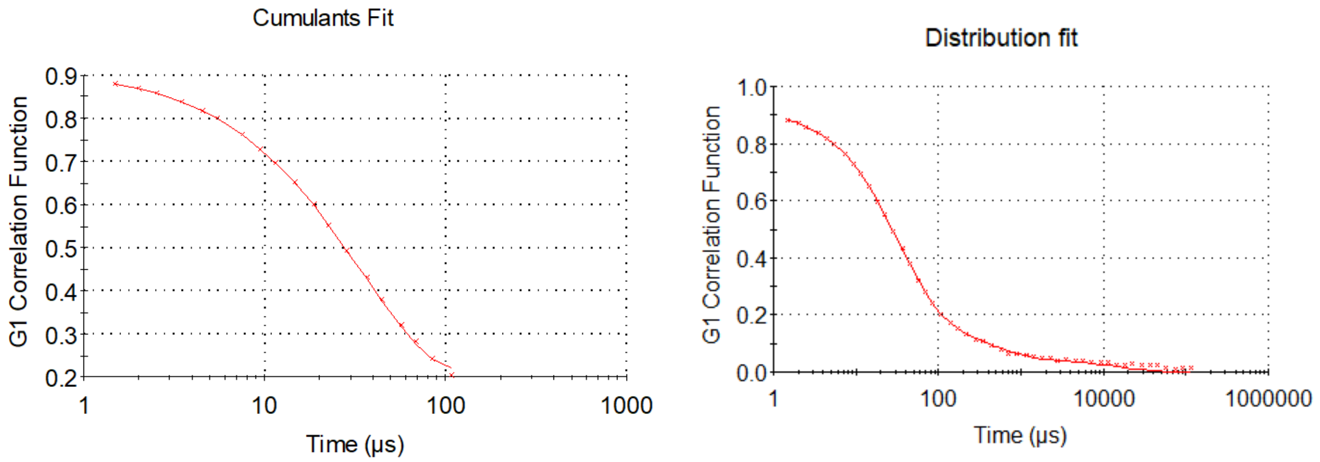

Figure S1. Dynamic light scattering (DLS) analysis of Frutalina (FTL). (a) Cumulant fit showing the average hydrodynamic diameter. (b) Distribution fit depicting the size distribution profile of FTL.

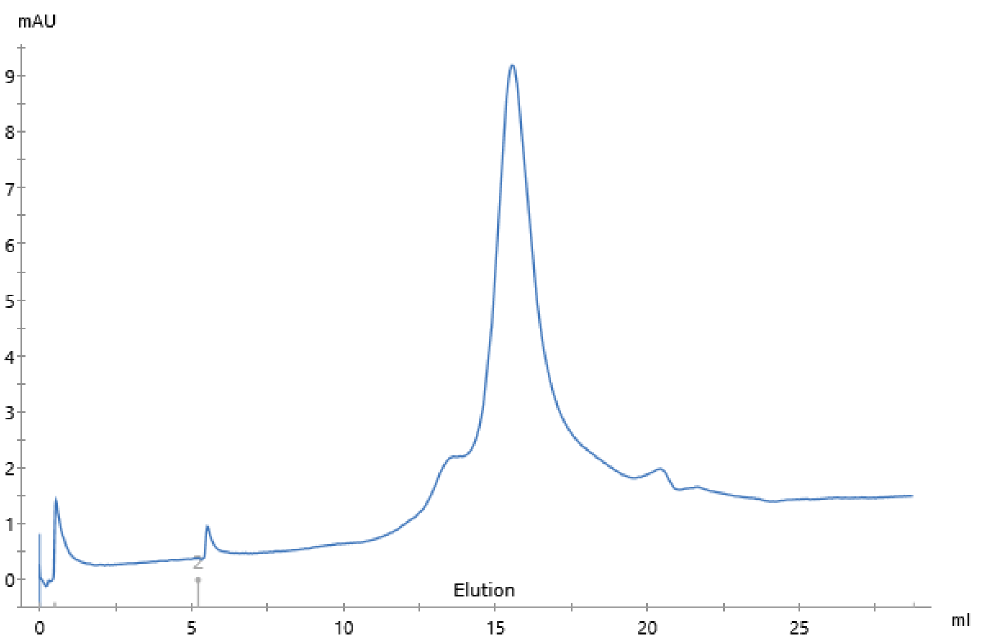

Figure S2. Size-exclusion chromatography (SEC) profile of Frutalin (FTL) obtained from the ÄKTA system. Absorbance at 280 nm (mAU) is plotted against elution volume (mL).

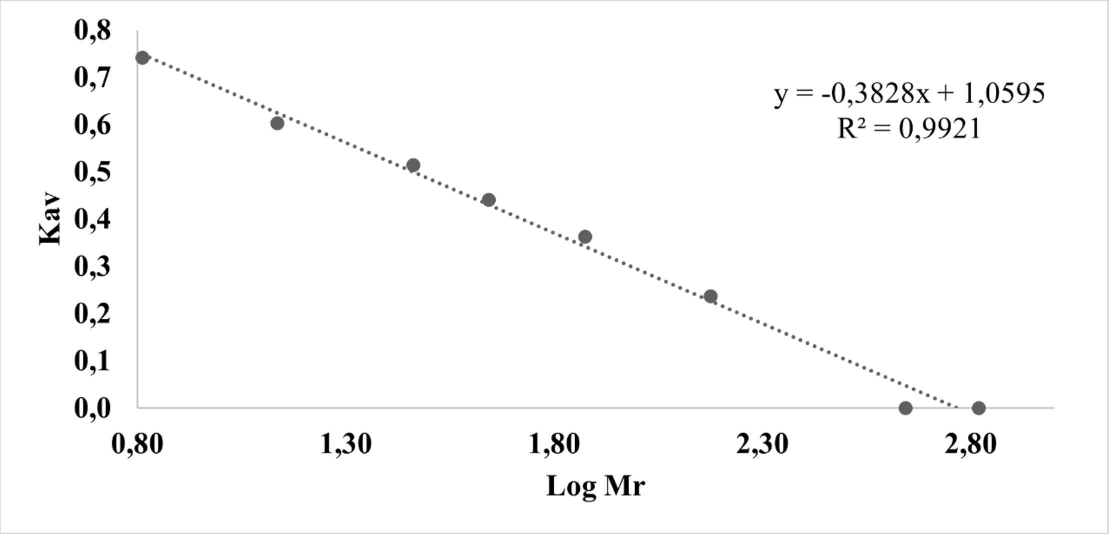

Figure S3. Calibration curve for size-exclusion chromatography (SEC). Partition coefficient (K_{av}) is plotted against the logarithm of the molecular weight (Log M_R) of standard proteins.

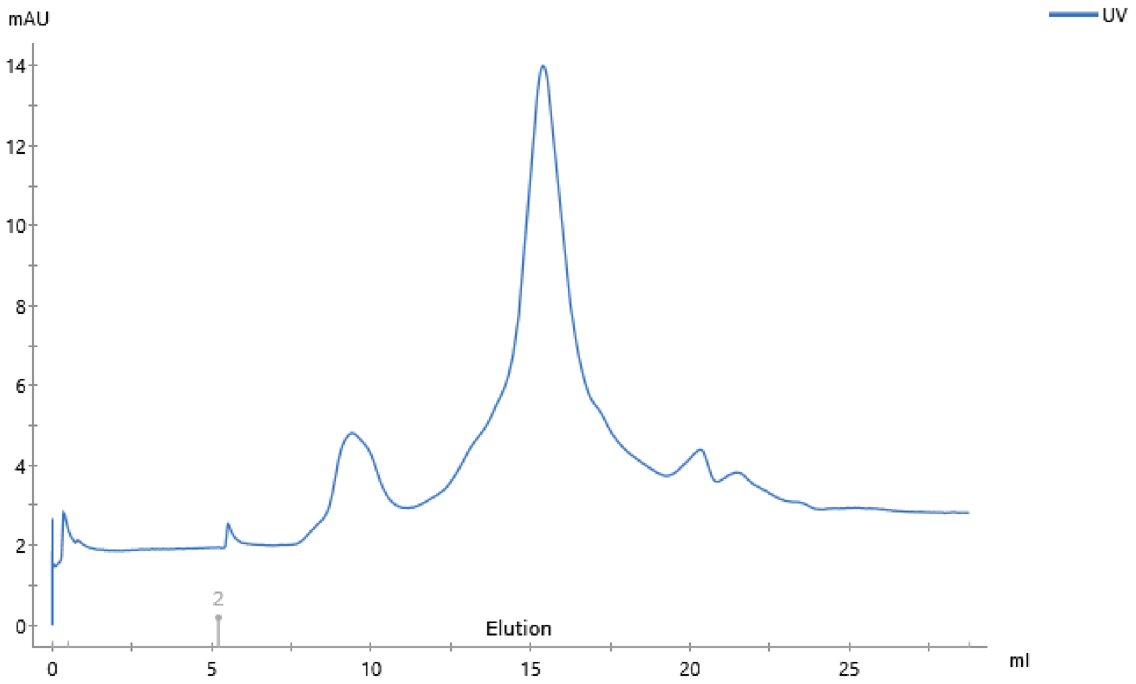

Figure S4. SEC analysis of the FTL–sIgA1 complex under competitive conditions. obtained from the ÄKTA system. Absorbance at 280 nm (mAU) is plotted against elution volume (mL). SEC was performed in the presence of 50 mM galactose. The complex largely dissociated, producing a peak at Ve15.4 mL corresponding to free FTL (~39 kDa apparent) and a peak at Ve 9.4 mL(~488 kDa apparent), consistent with residual high-avidity complexes.

Supplementary Tables

Table S1. Molecular parameters of standard proteins used for SEC calibration. Kav: partition coefficient calculated as Kav = (Ve – V_0)/(Vc – V_0), where V_0 is the column void volume and Vc is the column total volume; Ve: elution volume (mL); Mr: molecular weight (kDa); Log Mr: logarithm of molecular weight

Molecular pattern	Kav	Ve	Mr (kDa)	Log Mr
Thyroglobulin	-0,11094371	7,34	660	2,82
Apoferritin	-0,00273935	8,92	440	2,64
Alcohol dehydrogense	0,236269	12,41	150	2,18
Conalbumin	0,36296398	14,26	75	1,88
Ovalbumin	0,44035064	15,39	44	1,64
Carbonic anhydrase	0,51431311	16,47	29	1,46
Ribonuclease A	0,60265717	18,15 e 17,37	13,7	1,14
Aprotinin	0,74167922	19,79	6,5	0,81
